# What's law got to do with it Part 2: Legal strategies for healthier nutrition and obesity prevention

**DOI:** 10.1186/1743-8462-5-11

**Published:** 2008-06-05

**Authors:** Roger S Magnusson

**Affiliations:** 1 Faculty of Law, University of Sydney, Sydney, Australia

## Abstract

This article is the second in a two-part review of law's possible role in a regulatory approach to healthier nutrition and obesity prevention in Australia. As discussed in Part 1, law can intervene in support of obesity prevention at a variety of levels: by engaging with the health care system, by targeting individual behaviours, and by seeking to influence the broader, socio-economic and environmental factors that influence patterns of behaviour across the population. Part 1 argued that the most important opportunities for law lie in seeking to enhance the effectiveness of a population health approach.

Part 2 of this article aims to provide a systematic review of the legal strategies that are most likely to emerge, or are worth considering, as part of a suite of policies designed to prevent population weight gain and, more generally, healthier nutrition. While the impact of any one intervention may be modest, their cumulative impact could be significant and could also create the conditions for more effective public education campaigns. This article addresses the key contenders, with particular reference to Australia and the United States.

## Background

The potential for law to contribute to obesity prevention remains largely unrealised. Part 1 of this article drew on a hierarchy of determinants model to illustrate the different levels at which policy interventions can address obesity. Since there is a population-wide trend towards weight gain in Australia, policy efforts – including legal ones – need to engage with those factors that can plausibly be shown to influence the behaviour of wide segments of the population around nutrition, healthy eating, and an active lifestyle. A population health perspective is vital: "privatising" the obesity epidemic, confining it to the health care setting, and emphasising personal responsibility to the exclusion of population-wide interventions, will do little to reverse the trend.

Part 1 also outlined a framework for analysing the distinct contribution that law can make in obesity prevention and chronic diseases generally. The framework built on work by Professor Lawrence Gostin and others who have categorised the tools and strategies that law can offer in various ways [1,2]. Drawing on the typology of legal interventions outlined in part 1 (Figure 5), part 2 of this article will now review some of the most likely areas where legal interventions in support of obesity prevention deserve serious consideration. This part will focus primarily on energy intake issues relevant to obesity prevention, and more broadly, on healthy nutrition. Legal strategies to facilitate higher levels of physical activity in the population are also important but deserve separate and extended discussion [3-8].

## Effective structures for policy leadership and health governance

A comprehensive approach to population weight gain calls for policies that engage with a broad range of sectors and settings. These include the food system (primary production, manufacture, retail, catering and advertising of food), the built environment, transport and urban development, the education system (school curriculum, school food services), and taxation [9-11]. At present, health departments – the agencies that might be expected to "do something" about obesity – are overwhelmingly focused on the "sick care" system. Disease prevention and health promotion functions tend to lose out in the internal contest for resources. In 2005–2006, Australia spent $86.9 billion on health, but only 1.7% of this ($1.5 billion) was spent on public health [12].

Public health units lack the political clout that is necessary to achieve policy input and influence within other sectors. Urban planning decisions, for example, could facilitate physical activity in many different ways, but this is unlikely to occur without formal structures to ensure health input. In federal systems like Australia, control of relevant sectors is also fragmented between different tiers of government. For good reason, obesity prevention has been called "a brilliant test of political capability" and a "test for public health to think beyond its traditional boundaries" [13].

Arguably the most significant contribution that legislative or executive action could make to obesity prevention is by creating governance structures capable of coordinating a "whole of government approach" to obesity. At the broadest level, there have been calls for a separate Cabinet-level, Department of Public Health in each of the states and territories that is not dominated by the delivery of health care services and is capable of forging effective partnerships with other government agencies and the private sector [14]. In the United States, the Trust for America's Health has called for the Center for Diseases Control and Prevention (CDC) to act as a "command and control center" to coordinate states and agencies in a genuinely national policy response [15]. States have also begun experimenting with inter-departmental structures, such as the Interagency Obesity Council recently established in Texas, which brings together high-ranking officials from agriculture, health services and education to discuss nutrition and obesity prevention programs [16].

In Australia, Corbett argues that " [a] workable template is provided by the state-based Environment Protection Authorities", coupled with a Commonwealth agency providing national policy leadership and standards setting [14]. In the United Kingdom, Lang and colleagues have advocated a national policy council on food, nutrition and physical activity, with a statutory mandate, to provide independent advice and to ensure that government does not become captive to the entrenched cultures and agendas of existing departments. While not implementing policy itself, such an agency would examine links between the multiple sectors that impact on nutrition and physical activity, review policies, appraise solutions and monitor progress [17].

Significant work remains to be done to identify those institutional features that could best deliver policy improvements for obesity and chronic diseases across sectors and levels of government. The central choice is between "politically-owned" structures with cabinet-level leadership, or independent-of-government agencies with clear powers and cross-sectoral reach. The advantages of creating an independent Commission or policy council with a mandate to provide policy advice to government include its independence from the agendas and influence of existing departments, and its capacity to engage with industry, consumers and the NGO sector. Such an agency could also perform valuable functions that government itself might prefer not to perform directly; for example, evaluating industry practices and advocating prevention strategies in a robust manner. On the other hand, the challenge of influencing and coordinating policy as implemented by multiple agencies outside of the health sector remains. This cannot be achieved without political commitment at the highest levels, supported by aims and targets that bridge departmental and sectoral divides and bind agencies together in the common cause. Until chronic diseases are a Cabinet-level priority, there is unlikely to be sufficient executive authority to drive effective partnerships across departments, and into the broader community.

## Obesity prevention and the information environment

Information has an important role in obesity prevention. Commonwealth and state governments have an important health promotion function, as trusted providers of health information and advice. Law can serve obesity prevention by altering the information environment in several ways. These include by:

∘ generating information resources for use by governments and individuals;

∘ mandating the provision of information to consumers to facilitate healthier choices and, more controversially,

∘ restricting advertising to consumers.

### Using law to generate health information resources

Generating information about eating and physical activity patterns is a safe political response to calls for government action in response to obesity. In July 2006, the Minister for Health and Ageing announced a series of surveys to determine eating and physical activity patterns in children [18,19], and one of the non-contentious outcomes of the NSW Government Childhood Obesity Summit in 2002 was the NSW Schools Physical Activity and Nutrition Survey (SPANS). The SPANS survey confirmed that school age children in 2004 were more active and fitter than their counterparts in 1985 and 1997. But at the same time, rates of obesity and overweight had risen to nearly 25% [20].

There is no inherent reason why nutrition surveillance needs to be specifically mandated in legislation. What *is *needed is an adequate mandate and funding, and a consistent methodology for building a longitudinal picture of trends in food consumption and food ingredients [21,22]. Nutrition surveillance data are important for galvanising political commitment for public health policies, and for evaluating interventions taken at a population level, as well as primary and secondary prevention interventions.

In the United States, law has assisted the development of information resources in several additional ways. There are many examples of explicit Congressional mandates to various agencies to conduct specific research projects, and to develop specific data resources. For example, the *Children's Health Act of 2000 *directed the CDC to establish a national juvenile diabetes surveillance system [23]. The same Act authorised the Director of the National Institute of Child Health and Human Development to establish a consortium of federal agencies to plan and implement an ambitious, 25-year prospective study of environmental influences upon children's health and development [24]. Other examples include federal funding for a nationally representative study of foods purchased by school authorities participating in the federally funded National School Lunch program, in order to monitor the nutritional quality of these federally reimbursable meals [25].

A more controversial example of law's use to generate information resources is the extension of the notifiable diseases model to the context of chronic disease. Effective 15 January 2006, the New York City Department of Health and Mental Hygiene began a diabetes monitoring program, based on mandatory reporting of A1c (haemoglobin blood sugar levels) by the 127 laboratories connected to the Electronic Clinical Laboratory Reporting System. By making diabetes a notifiable disease, the Department will create a registry that maps the epidemiology of hyperglycaemia and monitors test results on a name-identifying basis [26]. Under a pilot project currently underway in the Bronx, patients with blood sugar levels above a certain level will receive information (on an opt-out basis) about the lifestyle changes required to reduce health risks. Treating physicians will also be alerted to patients whose diabetes is not well controlled and reminded of best practice recommendations [26-28]. This strategy for diabetes control will also provide opportunities for addressing obesity, a major risk factor.

Diabetes notification remains highly controversial [29-31]. On the other hand, the reporting of biophysiological markers for chronic disease (high blood sugar, blood cholesterol or blood pressure levels) could serve as a trigger for referral to individually-focused and supportive lifestyle interventions that could prevent further health deterioration and health care expenditure. The co-occurrence of various chronic diseases and risk factors among ageing populations means that risk factor surveillance could evolve as a powerful and efficient strategy for disease management and prevention [32-34]. Since high blood glucose levels and other risk factors for chronic disease disproportionately affect those of lower socio-economic status, notification could also help to address health inequalities [35]. In Australia, although governments pay for over two thirds of all health care expenditures, the risk factors for obesity and chronic disease are not communicable. In these circumstances, concerns about privacy, government lifestyle surveillance, and discrimination may support a right to opt out of mandatory reporting.

Schools are an important site for strategies targeting obesity in children. In 2007, the Trust for America's Health reported that twelve states had enacted legislation enabling or requiring schools to measure students' body mass index (BMI) and to report this information to parents [[15], pp27–28]. The assumption behind BMI screening is that it will identify school populations that are most in need of assistance, while reports to parents will prompt parents to make improvements in children's diet and levels of physical activity [36,37].

In 2003, Arkansas became the first state to adopt this strategy in an Act that also required schools to disclose funds received from competitive food and beverage contracts [38,39]. Although the BMI screening program received a positive evaluation after three years of operation [40], in 2007 the state legislature weakened the legislation, exempting senior students and permitting parents to veto assessment of their children [41,42]. Under a pilot program in California, BMI screening forms part of a non-invasive screening program for diabetes for 7^th ^and 8^th ^graders. Parents of children at risk of developing type 2 diabetes are informed of this and of the services available to assist with prevention. School districts are required to report to the legislature on screening statistics, and on the extent to which parents sought assistance for their children as a result of notifications [43]. The trend towards measuring BMI in schools has spread beyond the United States, with England and Wales announcing a similar policy [44].

### Mandating the provision of information to facilitate healthy food choices

Food labeling laws can play an important role in facilitating informed choices, both at point of consumption (in restaurants), and at point of purchase (in shops and supermarkets). In Australia, food regulation is shared between the Commonwealth and the States [**Appendix**]. The Food Standards Code, as developed by Food Standards Australia New Zealand (FSANZ), and approved by the Australia and New Zealand Food Regulation Ministerial Council, applies in all States and Territories, and covers a broad range of areas. These include labelling, food contaminants, additives, specific product standards relating to particular classes of foods and beverages, food safety requirements, and primary production standards. The direct and prescriptive regulation of food businesses is the norm across these areas. The critical point, however, is that FSANZ standards are designed to protect against the manufacture and sale of *unsafe *or *unsuitable *food. Provided it is not toxic or perished, the Food Standards Code does not impede the manufacture and sale of food based merely on its poor nutritional quality.

Nevertheless, the Food Standards Code does facilitate healthy food choices in several ways. For example, packaged foods are required to display a nutrition information panel indicating the amount of energy, protein, total and saturated fat, carbohydrate, sugars and salt per recommended serving and per 100 g or 100 ml [45]. "Truth in nutrient claims" is required. For example, "reduced fat" foods must be 75% or less of the fat content of the reference food (with at least a 3% fat per weight reduction for food); "low fat" foods must not contain more than 3% fat per weight, and "fat free" foods must not contain more than 0.15% fat per weight [46].

The Ministerial Council's *Policy Guideline on Nutrition, Health and Related Claims *[47] provides a framework for the development of a new standard for labelling of "functional foods" and for related health claims [48]. Reform in this area could make a substantial contribution to public health nutrition by protecting against deceptive and misleading claims, assisting consumers to choose foods that will contribute to a healthier diet, and stimulating industry to manufacture new "functional" foods by permitting them to inform customers of their possible health benefits. FSANZ's proposed regulatory framework is based on three categories of claims. "Nutrient content claims" are statements about the amount of energy or of a nutrient in a food. "General level health claims" and "high level health claims" both involve claims about the impact of consumption of a food or a property of the food on the healthy functioning of the human body, either specifically or at the population level. However, "high level claims" refer to a serious disease or a biomarker for a serious disease, defined as a condition that requires professional treatment or management – including obesity, but excluding overweight [49].

The new draft Standard restricts nutrition content and health claims for infant formula products, and in foods containing more than 1.15% alcohol (except with respect to the energy, carbohydrate and alcohol content of foods containing alcohol) [49]. Apart from this excluded category, FSANZ's proposed approach to health claims includes a scoring system to determine whether a product is eligible to carry either general or high level health claims. The scoring system is based on the food's nutrient profile, with points added for increasing amounts of energy, sugar, saturated fats and sodium, and points reduced for the percentage of fruits, vegetables, nuts, pulses, and fibre in the product [[49], pp99–105]. The intention is to prevent health claims being made for foods with high levels of risk increasing nutrients. The draft Standard sets out different conditions for making general, and high level, health claims. High level health claims require (convincing) scientific evidence of a diet-disease relationship [[49], pp29–34]. The Standard sets out a number of substantiated diet-disease relationships for which convincing evidence currently exists, together with the conditions that must be met and the statements that must be included on packaging for these claims [[49], pp81–85]. Similarly, the Standard sets out the descriptors that may be used to refer to a property in the food for the purposes of a general level health claim, and the conditions that apply to each type of claim [49].

Further reform of food labeling laws could significantly expand their utility in supporting healthier, informed choices. In their review of Australian and New Zealand research on nutrition claims and labels, Mhurchu and Gorton conclude that consumers find current labeling confusing and difficult to understand [50]. In a review of international studies, Cowburn and Stockley point to the difficulty consumers have in understanding the significance of nutrient information within the context of their overall diet. They argue that interpretational aids or benchmarks, such as verbal descriptors or guideline daily amounts, could assist consumers to place a product "into a total diet context" [51]. In a study across four European countries, Feunekes and colleagues confirmed the value of simple, front-of-pack labeling systems to empower consumers in making healthier choices quickly [52]. van Kleef and colleagues have argued that calorie labeling should also be included in any front-of-pack labeling scheme [53].

The "traffic light" system developed as a voluntary measure by the UK Food Standards Agency provides one response to these concerns. It provides a highly visible, front-of-package reference aid in the form of red, yellow and green labels and corresponding percentages that reflect levels of salt, sugar, fat and saturated fat in the food [54,55]. The simplicity of this system could make it highly useful as an educative tool, in real time, in supermarket isles. Industry claims that only diets – but not foods – should be described as "healthy" or "unhealthy" are hardly convincing, given the eagerness of the food industry itself to embrace "functional food" claims [56,57]. Some public health experts have gone further, arguing that traffic light dots (for example, a red dot to identify foods high in sugar, salt or fat) should "follow the food" wherever it appears: on television advertising, and on fast food packaging [58].

Food labels could also better support healthy choices by indicating, front-of-pack, the fat, sugar and salt levels of a portion or recommended serving as a proportion of the daily recommended intake for a normal adult eating a balance diet. A consumer's ability to make healthy choices might well be improved, for example, by knowledge that their "large meal deal" from a fast food chain delivered 52% and 77%, respectively, of average daily energy intake for men and women [59]. The same point applies to the value of fat, sugar and salt in a food item or portion, as a percentage of recommended daily levels. Currently the draft *Standard on Nutrition, Health and Related Claims *(Standard 1.2.7) permits voluntary labelling of percentage of daily intake information [[49], pp63, 114–116]. No form of front-of-pack nutrition labelling is required.

In the United States, the Nutrition Labeling and Education Act of 1990 introduced a standard label on mandated foods that permits comparison between the nutritional content of a food and the recommended daily values (RDVs) for nutrients including fat, saturated fat, sodium, carbohydrates, and fibre (although not protein or sugar) [60,61]. More extensive changes were introduced unsuccessfully into the Canadian House of Commons in 2003. Bill C-398 would have required meat, poultry and seafood packaging to display the number of calories and the amount of total fat, saturated fat, trans fat, cholesterol, sodium, carbohydrate, fibre, sugars, protein, iron, calcium and Vitamin A and C per serving, expressed as a percentage of recommended daily levels [62].

Recent decades have seen a trend towards greater consumption of "fast food" and food prepared away from home. In the United States, by 1994–1996, this category accounted for a third of calories eaten, with "away" foods containing more calories per eating occasion, more saturated fat and cholesterol, and less fibre per calorie than home foods [63]. In Australia, the number of fast food outlets doubled between 1992–2002, with Australian families purchasing fast food "on average once every three or four days" [64]. In view of these trends, one important question that arises is whether the opportunity to choose healthy foods deserves the status of a consumer right. State and Territory legislation requires the registration or licensing of food businesses, including restaurants, either by the local council (Vic, Qld, SA, WA, Tas), the State Food Authority (NSW), or the Chief Health Officer (ACT, NT, WA) [65]. These laws provide one mechanism for requiring – as a condition of license or registration – that restaurants offer real choice by including clearly identified item(s) on their menus that are low in salt, saturated fat, and sugar.

One alternative to mandating "healthy options" in restaurants is to extend the reach of labeling laws beyond pre-packed food to standardized food items regularly offered for sale in restaurants and fast food outlets. There is evidence that consumers vastly underestimate calories, fat, saturated fat, and salt levels in restaurant menu options, and also that nutritional information influences food choices to a degree that could avoid significant weight gain over time [66,67]. In December 2006, New York City pioneered this form of regulation by adopting regulations requiring food service establishments to prominently display calorie information for standardized menu items [68]. The new law was intended to apply to the estimated 10% of restaurants that offer "food menu items in portions that are standardized for size and content", and which (voluntarily) made calorie information publicly available on or after 1 July 2007 [68].

In September 2007, the law was struck down by the United States District Court, which ruled that federal legislation already prescribes the standards that restaurants *voluntarily *providing caloric information must comply with, and that this legislation pre-empted the New York City Board of Health's authority to impose new regulations in this area. Importantly, however, the Court made it clear that federal legislation would not pre-empt a law that would simply *impose *the calorie display rules on restaurants, provided that these rules were not limited to restaurants that *voluntarily *made calorie information available to customers [67,69]. Undeterred, the City re-introduced the law, making it applicable to the 2,400-odd restaurants in the city that belong to chains with fifteen or more outlets nationally. The new law will require calorie information to be displayed in a size and typeface that is at least as large as the description and price of the food item [70].

In September 2007, the California Senate voted to extend this menu labeling strategy through a Bill that also applied to restaurant chains with at least 15 outlets. The Bill would have required nutrition information to be printed on menus per standard menu item for: total calories, total grams of saturated and trans fat, total carbohydrates and milligrams of sodium [71]. In October 2007, however, the legislation was vetoed by Governor Schwarzenegger [71,72]. Similar requirements, contained in Canada's Bill C-398, would have applied to vending machine businesses and food businesses selling food to the public for immediate consumption [62].

In Australia, in the absence of all these kinds of strategies, the Heart Foundation's Tick Program has evolved as a market-driven scheme to assist consumers to identify the healthiest options from the range of supermarket products. Food products that are independently assessed to be in compliance with the Tick's nutritional standards receive the market advantages of the Heart Foundation's "Tick" logo, and the scheme is self-funded through licence fees based on gross sales of Tick-approved products. In August 2006, the tick was extended to everyday lunch and dinner meals eaten out [73]. More controversially, in February 2007, nine McDonald's meal combinations were awarded the Tick [74,75]. An important benefit of the Tick is the behind-the-scenes work which the Heart Foundation has carried out with industry to reduce levels of salt and saturated fat in food products, in order to achieve Tick certification [76].

### Food advertising: restricting information flows to consumers

Economic libertarians regard proposals to restrict food advertising with hostility and suspicion. One commentator writes:

The war on fat...reflects an anti-capitalist perspective that views people as helpless automatons manipulated into consuming whatever big corporations choose to produce. The anti-fat crusaders want to manipulate us too, but for our own good" [77].

What tends to be conveniently overlooked, however, is the extraordinary sums invested by food and beverage manufacturers in order to shape and influence consumer spending patterns (a fairly good proxy indicator of their real-world impact). In Australia, fast food companies spent over $110 million on mainstream advertising in 2005 [78]. Globally, in 2004, Pepsico and Coca Cola spent $1.7 and $2.2 billion on advertising, respectively, a total exceeding the World Health Organization's biennial budget [79]. In Australia, a recent study of free-to-air television between 7.00am–9.00pm showed that 31% of all advertisements were for food, the vast majority of which (81%) were for "foods high in fat, sugar and/or salt, and of low nutritional value" [80]. Take-away food, chocolate and confectionary dominated food advertisements, in that order. In the United States, a study comparing the nutrient values of the most heavily advertised foods against the recommended daily values (RDVs) that appear on food labels found that "general-audience composite food is particularly high in fat and sodium, whereas the child-audience composite food is especially high in sugar" [81].

In the United States, the First Amendment protects "freedom of speech" from legislative interference, although commercial speech enjoys a lesser degree of protection than social and political speech. In the *Central Hudson *case, the U.S. Supreme Court set out a four-part test for determining whether legislation that interferes with commercial speech is constitutional [82]. In summary, where government seeks to protect the public's health by enacting legislation that would have the effect of limiting commercial speech that is neither unlawful, nor misleading, courts will scrutinise whether the legislation "directly advances" the interest in public health, and "whether it is not more extensive than is necessary to serve that interest" [82]. This test has been applied in ways that protect the advertising of harmful or unhealthy products, including tobacco [83] and alcoholic beverages [84]. It is not clear that meaningful restrictions on the advertising of fast food or sugary beverages would be either lawful or politically feasible.

In Australia, there is no Bill of Rights and the impediments to legal constraints on advertising are political, rather than constitutional. Substantial restraints do exist in the case of tobacco [85]. So far, however, debate about regulatory approaches to food advertising has focused on limiting children's exposures. A growing literature demonstrates that foods high in sugar, salt and/or saturated fats are heavily advertised during children's television viewing hours [86,87], in a way that is grossly disproportionate to the small role these foods should play in a balanced diet [88]. Premium offers included in television advertisements during children's programming [89], food advertising to children in children's magazines and on associated food marketing internet sites [90,91], and food marketing targeting children in Australian supermarkets [92] are all intended to influence children's food preferences. Their real-world effect is doubtless to influence spending decisions by children, and to ratchet up the "pester power" influence of children upon adult purchasing decisions.

A growing body of evidence supports the conclusion that food marketing works. It shapes food preferences, beliefs about advertised products, and purchase requests [93]. While increased television watching correlates with increased consumption of highly-advertised foods and reduced intake of fruit and vegetables [94-96], there is plausible evidence that these outcomes are mediated by exposure to the advertising of these foods on television [93,97,98]. Commonwealth-funded television campaigns advocating fruit and vegetables, on the other hand, are a "drop in the ocean of food advertising" and cannot compete effectively against the diet-distorting effects of heavily promoted foods that are high in saturated fats, sugar and salt [99]. For all these reasons, public health advocates, and groups such as the Obesity Policy Coalition [100], strongly support regulatory constraints on foods high in sugar, salt and fat (HSSF foods) as part of a broader policy response to obesity prevention.

Australia's co-regulatory approach to advertising to children currently consists of the *Children's Television Standards *2005 (CTS) prescribed by the Australian Communications and Media Authority (ACMA) [101]. The CTS apply to children's programming that commercial broadcasters are required to broadcast during designated time bands. The CTS regulate premium offers such as toys sold with advertised products, and provide that "any reference to the premium must be incidental to the main product or service advertised" (CTS 20). Several studies have shown that this provision, and others in the CTS, appear to be routinely ignored by food advertisers [80,89,102]. The CTS are supplemented by a voluntary standard, the *Commercial Television Industry Code of Practice *[103], which applies to television advertising as a whole. The Code provides that food advertisements directed to children should not promote unhealthy eating and drinking habits, an inactive lifestyle, or contain misleading nutritional information (para 6.23). The ambiguity and non-functionality of current standards, as applied to food advertising to children, are discussed by Handsley, Mehta, Coveney and Nehmy [104].

A current review of the CTS has identified a range of possible regulatory reforms, although substantive changes seem unlikely [105]. This conclusion is reinforced by the weight given by ACMA to the impact that restrictions on food advertising to children might have on the profitability of children's programming [105]. In 2006, the Australian Democrats introduced a Bill that would have banned all food advertising to children, but this did not proceed [106].

Meanwhile, reforms are occurring in other countries. In the United Kingdom, the telecommunications regulator, Ofcom, has banned television advertising of foods assessed as high in fat, salt or sugar in or around programs aimed at children, or likely to appeal to children [107,108]. From 1 January 2008, these standards apply in respect of children 4–15 years. Since 1 July 2007, Ofcom's rules have also applied to paid-for internet ads, magazines and cinema ads [109]. The UK model depends upon nutrient profiling to identify foods high in sugar, salt and fat (HSSF foods) under a model developed for Ofcom by the Food Standards Agency. In Australia, the ACMA review has already dismissed this approach as an option, even though it contains an incentive for industry to improve the nutritional quality of foods in order to retain the right to advertise them to children [105].

Concern about food advertising to children is increasingly a global phenomenon. A global trend towards the development of self-regulatory standards by industry can be seen as one response to this rising concern. Barriers to stronger regulation include agreement on how to define HSSF foods, and the strength of the causal link between food advertising and obesity [110]. Any serious attempt at regulation in this area in future will also need to grapple with the increasing complexity of food advertising to children encompassing television, internet, mobile phones, as well as traditional print media [111].

## Economic policies for obesity prevention

### "Fat taxes" and public health nutrition

Obesity is an economic issue as well as a health issue. Economic policies aim to improve patterns of diet and physical activity not by dictating behaviour, but by changing the costs of behaviour [112]. In the United States, rising obesity rates over the past twenty years correlate with a rise in caloric intake through increased consumption of grains, sugars and fats [113]. The last twenty years have also seen enormous expansion in the commercialisation and globalisation of the world's food supply. In the United Kingdom, "more people now work in catering than in the entire rest of the food supply" [114]. Competition in the processed food market has led to increased serving sizes and sharp price differentials between processed, energy-dense and non-processed, less dense foods. There is growing evidence that fast food consumption is an independent factor in rising rates of weight gain, due to its energy density and impact on overall caloric intake [115]. In the American context, Finkelstein and colleagues note:

Between 1985 and 2000, the price of fresh fruits and vegetables, fish, and diary products increased by 188%, 77% and 56%, respectively, whereas sugar and sweets, fats and oils, and carbonated beverages increased at lower rates – 46%, 35%, and 20%, respectively. These trends in relative prices are consistent with rapid increases in the consumption of products made with added sugars and fats [113].

Taxes on foods that are high in fat, sugar and salt have been supported by the World Health Organization [116], and by nutrition experts [10,117,118], but rejected by the Australian government [119]. In the United States, however, by 2000, 19 states and cities had introduced taxes on snack foods, soft drinks and candy [120].

Public discussion of a "fat tax" assumes that categories of food defined as "unhealthy" (or specific ingredients in food, such as whole milk and cream) would be taxed, either for the purpose of diminishing consumption of those foods, or in order to fund education and public health programs encouraging healthy food choices and lifestyles. To the extent that the former aim was successful in modifying dietary preferences, funds to achieve the latter aim would be diminished [121]. An alternative approach, given the role of advertising in influencing consumers to purchase high-fat, high-sugar, high-salt (HSSF) foods, is to tax advertising of these kinds of foods [122], or, more ambitiously, to align such taxation with the health properties of advertised food and beverages [123].

Criticisms of fat taxes are many and varied. Drawing on a national dataset showing consumer food purchase patterns in the United States, Kuchler and colleagues concluded that a tax on salty foods would not significantly alter consumers' preferences for salty snacks, with the result that such a tax could raise significant revenue. They question, however, whether using these funds for mass education campaigns would be economically efficient, given the failure of such programs, hitherto, to reverse the trend towards population weight gain [121].

Mytton and colleagues point to the possible population health effects of diet substitution as a result of the imposition of fat taxes at significant levels. They argue that focusing the tax on the principal sources of saturated fat would reduce fruit and vegetable intake, resulting in greater consumption of salty foods to the detriment of population health overall [124]. Although a "best outcome" tax could, on their calculations, have a significant impact on mortality (preventing over 3,000 deaths annually from cardiovascular disease in the United Kingdom), the likely trade-off between reducing saturated fat in the diet, and reducing salt, suggests that food taxes would be better directed towards smaller, precise food categories [124]. Taxes on snack foods and sugary drinks, or more limited proposals such as the Obesity Policy Coalition's call for the removal of the GST exemption for highly-sugared cereals [100], could be consistent with this approach, and may even be politically feasible as a response to childhood obesity. But deeper benefits at the population level will require more than just a tax. They will require food manufacturers to actually improve the nutritional quality of their products [122,124].

Perhaps the most important concern is that "fat taxes" would be regressive, raising prices paid by the least well-off, without improving their diets [125]. For the most disadvantaged, food is a flexible budget category that must accommodate competing financial demands. While a tax might well reduce consumption of fatty, sugary foodstuffs by price-sensitive consumers, the unsolved problem is the affordability of (healthier) substitutes, not simply a reduction in consumption of fast foods [125]. There has been "disappointingly little discussion about what systems change would be necessary...to support a future state where...free market forces dictated prices of healthy food that were equal to or less than those of less healthy fare" [126].

At present, law's role within a reformed system of public health nutrition remains largely unexplored. While local approaches to improving public health nutrition are important and should be supported [127], these are unlikely to be sustainable without a government-led focus on reforming the "upstream" policies and conditions that have led to the status quo; that is, market forces that systematically encourage over-consumption of "empty" calories, at the expense of fresh fruit and vegetables [128]. Lang and Rayner have articulated an ambitious framework for re-shaping the roles of markets, government, and consumers in obesity prevention [123]. Similarly, Dowler argues that food should be re-conceptualised by government as a basic utility product – like water – preserving the role of the state as economic regulator to ensure quality and access. In the current environment, food has been "reconfigured as part of a consumerist commodity culture, with the responsibility shifted from the state to the individual" [129].

All of these approaches, if ever translated into practical terms, would likely challenge prevailing assumptions about government non-interference with the supply and demand forces in food markets. Key roles for economic policy could include the use of taxes and subsidies to promote the production and distribution of healthier foods at both the local and system-wide level; greater use of the government's own capacity as an employer and supplier of public services to improve standards of catering in schools, hospitals and all government departments; the creation of local area targets for obesity reduction and improved nutrition, together with incentives and resources for local governments and the private sector to achieve them [123].

### Workplace-based prevention and "wellness programs"

Although debate about a "fat tax" has focused on "junk" foods, tax policy can contribute in many other ways to obesity prevention. In the United States, employer-funded health plans provide health insurance coverage for some 70% of American adults [130]. Employer premium contributions are deducted from the taxable income of the employer; the employer's share of premium is also exempt from income and payroll tax payable by the employee, reducing the employee's tax liability to the extent of the contribution [131,132]. Although employer-funded coverage is not compulsory and operates on actuarial principles, there is growing evidence that investment in disease prevention and health promotion programs pay their way in reduced payments for medical care, reduced absenteeism and improved workforce productivity [133,134]. Employer-sponsored wellness programs are an important strategy for reducing health care costs and are expected to grow strongly in future [135,136].

The workplace is an important setting for addressing lifestyle health risks. In most organisations, individuals work at a finite number of geographic sites, share a common purpose and culture, and communication is relatively straightforward [[133], p306]. In the United States, there are at least two likely pathways for further expanding employer investment in health insurance coverage for wellness programs designed to address lifestyle health risks. Firstly, market driven expansion could be encouraged by partial tax relief for companies offering prevention programs meeting criteria for quality and effectiveness [137,138]. Secondly, wellness programs could expand through financial incentives imposed upon employees and purchasers of insurance directly.

In the United States, federal regulations permit premium discounts and other financial rewards (not exceeding 20% of the cost of employee coverage) to be given to individuals who meet the requirements of a "wellness program" that is reasonably designed to promote health or prevent disease [138,139]. Since higher BMI is associated with higher health care costs [140], individuals who control their weight and manage other lifestyle risk factors could reap the rewards in premium discounts and other benefits [125,141,142]. The prominence of personal responsibility as a driving value in American life suggests that financial incentives (and penalties) will play a greater role in health insurance coverage schemes in future [143,144].

In Australia, national health care coverage is assured through Medicare, and private health insurance is community rated [145]. Nevertheless, there is emerging evidence of the correlation between the lifestyle health risk levels of insureds, and the health care costs paid by private insurers [146]. Hitherto, the perceived benefits of social insurance have taken precedence over the argument that the cross-subisidies paid by low-risk to high-risk insureds actually eliminate any incentive for the latter to adopt healthier behaviours. Any change from a community-rated approach needs to take account of the possibility that premium discounts (in effect, taxes on bad habits) could worsen health inequalities by punishing those with the poorest access to the personal and community resources that permit wealthier, better educated people to lead healthier lives. Such an approach could also have a reverse effect: creating disincentives to private coverage, and increasing the burden on Medicare-funded services.

These concerns would not apply to such things as tax deductibility for gym memberships or for swimming pool passes. While tax benefits to encourage regular physical activity might have limited impact, they send out the right signals, and strengthen the market position of an industry that has its own incentives for encouraging regular exercise.

While employers in Australia do not offer health insurance to employees, the challenge remains for both private and public sector employers to become "health policy entrepreneurs" by taking greater responsibility for health in the workplace and acknowledging the contribution that they can make. While improved productivity, reduced absenteeism, and the reputational benefits of being an "employer of choice" could provide their own return on investment [147], tax incentives to encourage the introduction of "health and wellbeing" programs could further assist the trend towards these programs. As an important site for chronic disease prevention, workplace programs could address issues including smoking cessation, weight control, regular physical activity, nutrition, health screening, and diabetes management.

### "Tied grants" and other spending strategies for obesity prevention

The constitutional powers of governments to tax give them the resources to fund programs and to implement policies [1]. In most cases, Australian government programs are funded annually by appropriation bills in accordance with departmental budgets and there is no need for detailed legislation setting out spending objectives. On the other hand, funding conditionality and "tied grants" are powerful tools for driving policy "vertically" across different levels of government, or for ensuring compliance with policy objectives.

Legislation may not be necessary where stakeholders share similar objectives. In New South Wales, for example, a policy partnership between the NSW Department of Education and Training (which is responsible for State schools), NSW Health, the Catholic Education Commission, the Association of Independent Schools and the NSW School Canteen Association led to implementation of the NSW Healthy School Canteen Strategy. This was one of the tangible outcomes of the NSW Childhood Obesity Summit in November 2002. There are over 3,200 schools in NSW and nearly 1 million students. The Canteen Strategy categorises foods according to a traffic light system: "red" foods (not to be sold on more than two occasions per term); "amber" foods (should not dominate the menu and avoid large serving sizes); and "green" foods (foods that should fill the menu and should be promoted) [148]. In addition to the school canteen strategy, the NSW Government's Action Plan commits government to working with stakeholders to revitalise secondary school sports programs, and to providing professional support for implementing a Personal Development, Health and Physical Education syllabus [149].

In the United States, by contrast, law has been central in the ongoing struggle for control over school nutrition. In 2006, over 30 million children participated in the federally-funded school lunch program, and nearly 10 million in the school breakfast program [150]. Federal regulations contain nutrition standards for these federally subsidised meals [151]. Although certain "foods of minimal nutritional value" cannot be sold "in food service areas" during meal times, such foods *can be sold *elsewhere on campus, and in other respects, schools authorities have discretion to permit sale of "competitive foods" in competition with federally funded meals [152-154].

The nutritional impact of competitive food is exacerbated by the fact that core school funding in many states comes from local school districts, many of which have entered into "pouring rights contracts" with soft drink companies. These contracts involve lump-sum payments to the district in return for exclusive sales arrangements on school grounds, with additional payments being made when sales exceed the contractual quota [155]. Beverage companies also "advertise on scoreboards, in hallways, on book covers, and elsewhere" [156]. To the extent that schools (or school districts) become dependent on canteen and vending machine revenues for funding of school food service programs, extracurricular activities, or even core programs, or are captured by exclusive contracts with food and beverage manufacturers, legislation may be necessary to ensure implementation of healthy nutrition policies.

At federal level, under the Child Nutrition and WIC Reauthorization Act of 2004, local education agencies that participate in the school lunch and breakfast programs are required to develop "local wellness programs" in order to qualify for federal funding [157]. "Local wellness policies" must include goals for nutrition education in the curriculum, the integration of physical activity across curricula throughout the school day, and must comply with nutrition guidelines for federally-subsidised school meals. A recent Institute of Medicine report on nutritional standards in schools classifies "competitive" foods into two tiers, and provides guidance to local agencies implementing school nutrition policies [158].

In addition, the Child Nutrition Promotion and School Lunch Protection Act currently before the US Congress aims to address the poor nutritional content of competitive foods by requiring the Secretary of Agriculture to issue regulations re-defining (and broadening) the prohibited category of "food of minimal nutritional value". These regulations would apply throughout the school day, to all food sold anywhere on the school campus, thereby improving the quality of foods sold in competition with federally-funded meals [159]. These laws would operate in addition to voluntary guidelines for competitive food and beverage marketing to schools, as agreed to by three large beverage makers and five snack food manufacturers, and brokered by the American Heart Foundation and the William J. Clinton Foundation [160,161].

Funding conditionality and "tied grants" are important strategies available to federal governments in countries like Australia and the United States where there is fiscal inequality between the federal and state levels. Under the *Schools Assistance Act *2004 (Cth), Commonwealth funding to state government schools is conditional upon each school providing a minimum of two hours per week of physical activity in the curriculum for primary and junior secondary schools [162]. In addition, the Public Health Outcome Funding Agreements (PHOFAs) provide Commonwealth funds for the States to spend in public health priority areas [163]. The priority areas under the current agreements (2004/5 to 2008/9) are communicable diseases, cancer screening and health risk factors. The performance indicators for health risk factors focus on alcohol, tobacco, and sexual and reproductive health. An important opportunity exists for the Commonwealth to use the PHOFAs to commit the states to comprehensive strategies for obesity reduction.

Conditionality could be used in a variety of other ways. For example, Commonwealth and state governments could – as landlord and employer – create accreditation standards for catering and food services in government buildings and agencies [2,164]. Important opportunities exist at local government level to improve the physical environment to facilitate walking and cycling, and through point-of-service prompts to walk or use stairs. Funding strategies (matching funds, conditional grants) could play an important role in creating incentives for local governments to spearhead environmental changes that are responsive to local conditions [165].

### Provider-based interventions: funding preventive clinical services

A final category of economic policies for obesity prevention focuses on the clinical health care system as an important setting for assessing obesity and related lifestyle risks, and for making "lifestyle prescriptions" (see Figure 2 in part 1 of this article). Unlike "upstream" strategies that engage with the environmental, social and economic determinants that apply broadly across the population, provider-based strategies focus on those who come in contact with the health care system, usually because they are already sick. Extending the "product line" of the health care system into disease prevention is an important part of a comprehensive approach to chronic disease.

Recent Australian initiatives in this area include the Commonwealth-funded *Lifescripts *program that is being implemented through local divisions of general practice. Based on the SNAP framework developed by the Royal Australian College of General Practitioners [166], *Lifescripts *encourages general practitioners to assess patients for lifestyle risk factors and to make written lifestyle prescriptions [167]. In Australia, the Medicare system now covers a range of allied health services provided by diabetes educators, dieticians, exercise physiologists and a range of other allied health professionals to patients requiring multidisciplinary care as part of an Enhanced Primary Care (EPC) plan [168-170]. Recently Medicare eligibility was relaxed to permit General Practitioners to refer patients with type 2 diabetes to access these services, as part of a GP Management Plan (MBS items 81100–81125) [171].

However, these initiatives are still part of the "sick care" system: their focus is not preventive. The challenge remains to create incentives for primary health care workers to implement *Lifescripts*, and to reduce lifestyle risk factors before disease manifests. One step in this direction is a new Medicare Benefits Schedule (MBS) item to encourage general practitioners to provide a comprehensive health check on patients around 45 years of age who present with identifiable risk factors for chronic disease (MBS Item 717) [171]. Recent legislative changes also permit private health insurers to offer "general insurance" coverage, including services that are intended to "manage or prevent a disease, injury or condition" [172].

## Direct, prescriptive regulation of businesses, organizations and individuals: the emerging law of public health nutrition

There are many other opportunities for addressing the determinants of obesity and chronic disease through prescriptive laws that directly regulate the activities of businesses and other organisations. As noted above, alarm at child obesity in the United States has resulted in a range of prescriptive interventions that include: imposing nutrition standards on foods sold in schools, introducing physical education requirements in the curriculum, and placing restrictions on when competitive foods can be sold in schools [173]. Texas law, for example, requires primary school children to engage in moderate or vigorous physical activity for at least 30 minutes daily, with annual physical fitness assessments, and ongoing assessment of the relationship between obesity, school attendance levels, and academic achievement in each school district [174]. In New Jersey, all candy, all products containing trans fats, and food and beverages listing sugar as the first ingredient are banned from sale on school property during the school day [175]. State and local school nutrition regulations include caps on calories and fat per serve [15]. In California, this has led snack food makers to shrink portion sizes and to bake rather than fry. But it has not yet eliminated junk food from schools, where it competes with federally subsidised school meals [176].

In Australia, as noted above, although the Food Standards Code reflects a prescriptive approach to regulation of the food industry, the focus has hitherto been on food safety and, as regards food labelling, consumer empowerment, as distinct from the nutritional quality of food [**Appendix**]. One area where the law of public health nutrition could develop further, however, is through the imposition of nutritional standards for the food industry as a whole. "Harm reduction", as applied to the regulation of nutrition, is still a novel theme, although this could well change in future. New York City has banned artificial trans fats in restaurant cooking in order to reduce the population risk for heart disease [177,178], and this measure is spreading at state and local level across the United States [179].

In 2006, a National Collaboration on Trans Fats led by the National Heart Foundation of Australia, the Dietitians Association of Australia, the Australian Food and Grocery Council, and Food Standards Australia New Zealand (FSANZ), was established to propose initiatives to reduce trans fats in food sold in Australia [180]. In 2007, FSANZ reported that trans fats represent 0.6% and 0.7%, respectively, of energy intake in Australia and New Zealand, well below the World Health Organization's goal of 1% [181]. FZANZ opposes labelling of trans fats in the belief that this could lead consumers to choose products that contain higher levels of saturated fats, even though they might be lower in trans fat [181]. In the absence of mandatory labelling, consumers are completely reliant on the extent to which the National Collaboration is successful in negotiating voluntary reductions.

Although the removal of trans fats is unlikely to impact on obesity levels, it is important to consider the role that nutrition regulation could play in reducing risk factors for chronic disease across the population. In 2006, the American Medical Association called on the Food and Drug Administration to revoke the current status of salt as "generally recognised as safe" and to regulate it as a food additive, imposing limits for added salt in various categories of processed and fast foods, and requiring warning labels for foods considered high in salt [182]. Since over three quarters of salt intake in western societies comes from processed and restaurant foods, mandatory limits on salt content across different product categories – with warning labels flagging the high-salt content of any exceptions – would be the most efficient way of achieving population-wide reductions in blood pressure and cardiovascular disease [183,184]. Less dramatically, legislation requiring highly salted products to be labelled as such would likely result in the disappearance of many of those products from the market, and a corresponding increase in reduced salt variants [185]. The World Health Organization has acknowledged that regulatory approaches are justified in some countries "where for years voluntary approaches have proved ineffective" [[186], p47].

## Conclusion

Obesity is increasingly being seen as a socially credible threat to health, although there is vigorous debate about its causes, and who bears responsibility for taking action. The failure to look beyond the frame of "personal responsibility" and the internal resources of individuals for solutions to what is a population-wide trend, does not bode well for effective public policy-making in future. This is compounded by the failure of current laws and policies to adequately facilitate informed choice and personal responsibility for healthy eating, as illustrated by food labelling laws. In Australia, and elsewhere, it will be important to document what policies are failing [187], to encourage governments to adopt a population health perspective, and to experiment with novel, yet plausible strategies.

No government has yet been successful in reversing the trend towards population weight gain [123]. As argued in part 1 of this article, the most promising models are those that recognise the subtle, yet pervasive impact of the economic, social and environmental determinants of modern "obesogenic environments" [11,123,164,188-190]. A regulatory approach could make a substantial contribution to addressing these structural determinants, although this will require governments to adopt a clear theoretical approach to the problem, to exhibit a degree of bravery and to demonstrate a commitment to outcomes that extend beyond the usual timeframes that operate in politics [191]. While the impact of any one "structural" intervention is likely to be modest, their cumulative impact could be significant, and could create the conditions for more effective individually-oriented policies, including education and health promotion [188].

The opportunities for legal interventions reviewed in this article are only a beginning. Some interventions, including law's role in encouraging breastfeeding [192,193], and in re-shaping the urban and built environment to better facilitate physical exercise and access to healthy food [3-8], have not been discussed. Evidence of the higher cost of healthy food in remote locations [194], and of the link between a healthy diet and proximity to healthy food in urban environments [195-197], suggest a strategic role for state and local governments, and planning authorities, in improving the landscape of public health nutrition. For example, as discussed by Hodge, Garcia and Shah, zoning laws have been used at municipal level in the United States to ban or to limit the density of fast food restaurants [198,199]. In February 2008, the New York City Council increased the number of permits for sidewalk carts selling fresh fruit and vegetables in poorly-served (poor) neighbourhoods [200,201]. In the United States, a range of local food initiatives are emerging that blend concerns about environmental sustainability, health, and the local economy [202,203].

Obesity and chronic disease are inter-generational challenges, and much of the law in this area remains to be written. Progress is likely to be incremental [191]. For all its commitment to libertarianism and free markets, many innovative responses to obesity are being trialled at different levels of government in the United States [198]. This article has not attempted to specifically critique the policy responses of Australian governments to obesity. The challenge remains, however, to act cooperatively across sectors and levels of government and to realise the potential of law as a policy tool, together with other strategies, to wind back the impact of overweight and obesity on chronic disease in Australia.

## Appendix: A summary of food regulation in Australia

In Australia, the *Food Standards Australia New Zealand Act *1991 (Cth) establishes Food Standards Australia New Zealand (FSANZ) as an independent authority with a statutory mandate to develop standards in a broad range of areas relating to the composition, production, handling and sale of food (ss 13, 16). This statutory mandate gives FSANZ control over food labelling, promotion and advertising, and authorises FSANZ to develop standards to prohibit the sale of food except on the conditions specified (s 16(1)(ca), 16(1)(d)). In developing standards and guidelines (known as "food regulatory measures", in the Act), the statutory objectives of FSANZ are to protect public health and safety, to facilitate informed choices through the provision of information, and to prevent misleading and deceptive conduct, although FSANZ must also have regard to best available scientific evidence in making risk assessments, and "the desirability of an efficient and internationally competitive food industry" (s 18).

Although FSANZ is empowered to initiate proposals for the development of food regulatory measures itself, as indicated in a three year forward plan published annually (s 20), executive control over food regulatory policy is maintained by the Australia and New Zealand Food Regulation Ministerial Council (ANZFRMC), which can adopt, amend, request review of, or reject draft standards (ss 84–87). The Ministerial Council, which is comprised of Commonwealth, State, Territory and New Zealand Health Ministers, also develops policy guidelines which FSANZ publishes and must take into account in developing draft food regulatory measures: (see [204]). Once accepted by Council, new standards are published and become part of the *Australia New Zealand Food Standards Code*, a comprehensive set of standards covering food constituents, manufacturing requirements, additives, product standards for key classes of food and beverages, food safety practices, and primary production standards.

Under the Food Regulation Agreement, signed in December 2002 between the Commonwealth, State and Territory heads of government, the States and Territories agreed to implement the Food Standards Code as a cooperative national system for food regulation. This has been achieved under State and Territory Food Acts which require compliance with the Code: Food Act 2003 (NSW) s 21; Food Act 1984 (Vic) s 16; Food Act 2006 (Qld) s 39; Food Act 2001 (SA) s 21; Health (ANZ Food Standards Code Adoption) Regulations 2001 (WA); Food Act 2003 (Tas) s 21; Food Act 2001 (ACT) s 27; Food Act 2004 (NT) s 20.
